# Improvement of oxidative stability and shelf life of Beluga (*Huso huso*) fillets using nanocomposite films constituted with *Prunus armeniaca* L. gum exudates (PAGE), Tragacanth gum (TG), fucoidan, and zinc oxide

**DOI:** 10.1016/j.fochx.2025.102842

**Published:** 2025-07-29

**Authors:** Sahba Bahrami Freadooni, Leila Nateghi, Ladan Rashidi

**Affiliations:** a Department of Food Science and Technology, VaP.C., Islamic Azad University, Varamin, Iran; b Research Center of Food Technology and Agricultural Products, Standard Research Institute (SRI), P.O. Box 31745-139, Karaj, Iran

**Keywords:** Beluga (*Huso huso*) fish fillet, TG and PAGE, Fucoidan and zinc oxide, Oxidation and microbial deterioration

## Abstract

The high perishability of fish products, particularly Beluga (*Huso huso*) fillets, poses major challenges in maintaining quality during storage. This study aimed to develop bio-based nanocomposite coatings to reduce spoilage and extend shelf life. Fillets were coated with *Prunus armeniaca* gum (PAGE) or tragacanth gum (TG) (1 % *w*/w), enriched with fucoidan (0.5 % w/w) and zinc oxide nanoparticles (ZnO, 0.5 % w/w). Acidity, pH, peroxide value (PV), total volatile base nitrogen (TVBN), thiobarbituric acid reactive substances (TBARS), color factors, and microbial properties were assessed during 14 days of storage. Fish fillets coated with nanocomposite films containing 1 % PAGE, 0.5 % fucoidan, and 0.5 % ZnO, as well as 1 % TG, 0.5 % fucoidan, and 0.5 % ZnO, showed the lowest pH, acidity, PV, TVBN, and TBARS compared to the control. These coatings improved redness, texture, and reduced bacterial count, confirming the potential of natural nanocomposites as effective edible coatings for preserving fish products.

## Highlights

• Gum-based films with fucoidan and ZnO preserved beluga fish fillet freshness.
• Natural coatings reduced oxidation and spoilage in stored beluga fish fillets.
• Fucoidan-ZnO films improved shelf life and quality of beluga fish fillets.
• Edible coatings slowed microbial growth and oxidation in beluga fish fillets.
• Nanocomposite coatings maintained texture, color, and safety of fish fillets.

## 1 Introduction

Over the past few decades, significant efforts have been made to produce healthy food products Sheybani, Rashidi, Nateghi, Yousefpour, & Mahdavi, 2023a). Fish has been considered as one of the important parts of the human diet due to its containing valuable nutritional components (Asghari, Shabanpour, & Pakravan, 2014; Ei Sheikha et al., 2022). Fish contain high-quality proteins with essential amino acids and are a good source of B-vitamins; fatty fish additionally provide vitamins A and D (FAO, 2020). Fish lipids are rich in EPA and DHA—long-chain omega-3 PUFAs known for their anti-inflammatory, cholesterol-lowering, and cardiovascular-protective properties (Maulu, Nawanzi, Abdel-Tawwab, & Khalil, 2021; Cheng et al., 2023). They participate in important body functions and indicate various biological activities such as anti-inflammatory activity, and lowering the blood cholesterol level, leading to a reduction in heart attacks and strokes (Maulu et al., 2021). Furthermore, oils that are high in omega-3 fatty acids play a vital role in ensuring optimal brain function (Sheybani, Rashidi, Nateghi, Yousefpour and Mahdavi, 2023a, Sheybani, Rashidi, Nateghi, Yousefpour and Mahdavi, 2023b; Yousefi, Nateghi, & Rashidi, 2024). Also, the oils rich in PUFAs have vitamin E which can act as a strong antioxidant (Asghari et al., 2014; Yousefi et al., 2024). Sturgeons are types of fish with highly valuable economic and nutritional indexes. Among them, juvenile Beluga (*Huso huso*) has an important role in supplying meat and caviar (Asghari et al., 2014). Beluga has been reported to have a high growth rate and also high adaptability to unfavorable rearing conditions. The high levels of polyunsaturated fatty acids (PUFAs) found in Beluga, similar to other fish species, render it susceptible to biochemical processes, including oxidation (Vital et al., 2018). Moreover, fish texture can deteriorate through protein degradation or enzyme-mediated decomposition and microbial growth (Sayyari, Rabani, Farahmandfar, Esmaeilzadeh Kenari, & Mousavi Nadoshan, 2021; Vital et al., 2018).

Deterioration of fish texture and quality of other food products through oxidation, enzymatic degradation, and microbial growth can be controlled using appropriate coating and covering methods (Geng et al., 2024; Gohari, Nateghi, Rashidi, & Berenji, 2024; Shirai, Baú, Zanela, & Pimentel, 2025; Vital et al., 2018). Gums, microfibrillated cellulose, carboxymethyl cellulose, chitosan, and protein isolates have been investigated to inhibit the fish, meat, and fruits decay (Ei Sheikha et al., 2022; El Sheikha et al., 2022; Geng et al., 2024; Shirai et al., 2025; Zhang et al., 2023). *Prunus armeniaca* L. gum exudates (PAGEs) which are collected from apricot trees, have been widely used in drug and food applications (Bahrami, Nateghi, Rashidi, Nobandegani, & Ghorbanpour, 2025; Gohari et al., 2024). PAGEs can be considered as fat replacers, emulsifiers, stabilizers, thickeners, and the encapsulating agents (Gohari et al., 2024). In a study, sodium caseinate and apricot tree gum were used to generate nanocomplexes to preserve the conjugated linoleic acid (CLA) (Gohari et al., 2024). It was reported that nanocomplex of PAGE containing CLA exhibited higher oxidative the stability than free CLA during 30 days of storage (Gohari et al., 2024).

Tragacanth gum (TG), a natural dietary fiber, has been reported to possess film- and coating-forming abilities. It is obtained from plants of the *Astragalus* genus, which are native to the Middle Eastern region (Nejatian, Abbasi, & Azarikia, 2020). Tragacanth gum (TG) is composed of two main fractions: tragacanthin, which is highly water-soluble, and bassorin, which has low solubility. While TG alone has limited techno-functional properties, its application in composite formulations has been reported to significantly enhance performance (Nejatian et al., 2020). There are other reports that using gums can inhibit the intensive deterioration of fish components. In a study by Yang et al. (2022), the myofibrillar protein oxidation of rainbow trout was controlled by development of three-layer coatings fabricated by tape casting procedure using flaxseed gum, chitosan, and flax seed gum incorporated with eugenol and laurel essential oils. It was reported that composite coatings decreased the declining rate of antioxidant enzyme activity, thus delaying protein oxidation (Yang et al., 2022). Basil seed gum and *Lepidium perfoliatum* seed gum were used with *Foeniculum vulgare* essential oil as nanocomposite edible coating on the shelf life of *Oncorhynchus mykiss* fish fillets during the storage (Sayyari et al., 2021). Although total volatile basic nitrogen (TVB-N), peroxide value (PV), thiobarbituric acid (TBA), and total bacteria counts (*Staphylococcus aureus*, *Escherichia coli*, and *Pseudomonas aeruginosa*) were enhanced during the storage for coated samples and non-coated sample, the lipid oxidation and microbial count of fish fillet were significantly controlled (Sayyari et al., 2021).

Xanthan gum was used to protect the Mackerel Tuna (*Euthynnus affinis*) fillets against the oxidation and microbial spoilage (Ei Sheikha et al., 2022). Xanthan gum coating led to significant protection of fish fillets against oxidation and microbial spoilage (Ei Sheikha et al., 2022). In other studies also have mentioned that natural biopolymer based coatings such as carboxymethyl cellulose and microfibrillated cellulose can increase the shelf life of chicken meat and banana during the storage (El Sheikha et al., 2022; Geng et al., 2024).

Fucoidan is a sulfated polysaccharide predominantly found in brown macroalgae, such as *Fucus vesiculosus* and *Laminaria japonica*, known for its diverse biological activities, including antioxidant, antiviral, and anti-inflammatory effects (Oliyaei, Altemimi, Abedi, & Hashemi, 2025).

Marine algal polysaccharides (MAPs) are extracted from green, brown, and red algae. The biomass of these algae comprises compounds like β-carotene and polysaccharides such as ulvan, fucoidan, laminarin, mannitol, and cellulose, contributing to their functional properties in various applications (Li et al., 2023; Pradhan, Bhuyan, & Ki, 2023). MAPs are components of the cell wall that help conserve energy and protect algae from harm (Qiu et al., 2024). *Sargassum ilicifolium* is a kind of brown macroalgae rich in fucoidan which encompasses the cell wall structure (Flores-Contreras et al., 2023). The main monosaccharide of fucoidan is fucose which distinguishes it from other polysaccharides (Bahrami et al., 2025). Fucoidan is a sulfated polysaccharide mainly made up of α-(1 → 3)- and/or alternating α-(1 → 3)- and α-(1 → 4)-linked L-fucopyranose units. This structural arrangement enhances its significant antioxidant and antimicrobial properties, which allows fucoidan-rich marine algal polysaccharides (MAPs) to serve effectively as edible coatings that prolong the shelf life of different food items (James, Long, O'Hara, Williams, & Moghaddam, 2024; Lan, Liu, Li, & Wu, 2024).

This study aimed to examine the effects of PAGEs, TG, and fucoidan along with zinc oxide on the shelf life of Beluga fish fillets. The considered goal will be achieved by wrapping the fish fillets with composite films containing TG or PAGEs, with and without fucoidan, and with and without zinc oxide. The regular corresponding quality indicators including color, pH, acidity, peroxide value, TVB-N, TBA, microbial counts (*Enterobacteriaceae, S. aureus*, and total aerobic counts), mold and yeast counts, texture, and sensory of covered/coated fish fillets were measured throughout the storage period. This study tried to discover new coating and film formulations to extend the high-sensitive marine products.

## 2 Materials and methods

### 2.1 Materials

Beluga fish (*Huso huso*) were procured from a local fish market located in Babolsar, Iran. A total of 10 fish were collected within 12 h after harvesting. These fish were farmed, exhibiting an average weight of 6.5 ± 0.3 kg and an average length of 85 ± 5 cm. Immediately following collection, the fish were packed in crushed ice and transported to the laboratory under chilled conditions (0–4 °C). Upon reaching the laboratory, the fish were rinsed with cold water, beheaded, and de-tailed. The abdominal cavity was meticulously cleaned, and the fish were manually filleted using sterile stainless-steel knives. The dark and light muscle portions were not separated, and the fillets were cut into uniform pieces measuring approximately 5 × 5 × 1 cm^3^. These fillet pieces were stored at 4 °C and utilized within 2 h for subsequent processing, which included edible coating and quality analyses. MAPs rich in fucoidan were extracted and purified in our previous study (Bahrami et al., 2025) and described in section 2.2. Tragacanth gum (TG) with an average molecular weight of 8.4 × 10^5^ g/mol was obtained from a local store in Tehran, Iran. *Prunus armeniaca* L. gum exudates (PAGE), with an average molecular weight of 5.69 × 10^5^ g/mol, were collected from apricot trees in Jupar (Mahan District, Kerman County, Kerman Province, Iran). Zinc oxide nanoparticles (ZnO, ≥99 %, Sigma-Aldrich, Product No. 544906) were used in this study. Microbiological media including Plate Count Agar (Sigma-Aldrich, Product No. 70152), Mueller Hinton Broth (MHB, Product No. M3699), Violet Red Bile Dextrose Agar (VRBD, Product No. 70185), and Yeast Extract Glucose Chloramphenicol Agar (YGC, Product No. Y2377) were purchased from Sigma-Aldrich (St. Louis, USA). Bacterial strains *Escherichia coli* (ATCC 25922) and *Staphylococcus aureus* (ATCC 25923) were also sourced from Sigma-Aldrich.

### 2.2 Extraction of fucoidan

Marine algal polysaccharides (MAPs) that are abundant in fucoidan were obtained through a subcritical water extraction (SWE) technique, as outlined by Bahrami et al. (2025), with adjustments made to enhance yield and purity. A SynthWave batch reactor system (Milestone, Bergamo, Italy) featuring a 200 mL reaction chamber was employed for the SWE procedure. The brown seaweed biomass underwent washing, drying, and milling into a fine powder, which was then combined with distilled water in a 1:10 (*w*/*v*) ratio. To remove dissolved oxygen and avert the oxidation of thermolabile compounds during the heating phase, the reactor was purged with nitrogen gas for 5 min. The mixture was heated to 140 °C under a constant pressure of 50 bar for 12 min, which allowed for the effective release of sulfated polysaccharides while reducing degradation. Following extraction, the hot slurry was filtered through a 0.45 μm membrane filter. The solid residue, which included fucoidan and co-extracted proteins, was preserved for further purification, while the aqueous filtrate, primarily consisting of low molecular weight compounds and pigments, was discarded. The collected solid was then suspended in 400 mL of a 2 % (w/v) calcium chloride (CaCl₂) solution and gently stirred at 70 °C for 30 min. This process promoted the selective precipitation of alginate and residual proteins, as calcium ions create insoluble complexes with alginate. Subsequently, the suspension was centrifuged at 10,000 ×*g* for 10 min at 25 °C, and the resulting supernatant, now rich in fucoidan, was gathered. The fucoidan-enriched extract was dried under a gentle airflow at 35 °C or through lyophilization for further analysis. The purification of the extracted fucoidan was conducted following the method described by Bahrami et al. (2025). The extracted polysaccharide underwent dialysis using a dialysis cassette with a molecular weight cutoff (MWCO) of 10 kDa. The pH of the obtained filtrate was adjusted to 6.7 and it was dialyzed again using the same dialysis cassette.

### 2.3 Preparation of fish treatments

Fish treatments were made by wrapping fish fillets using casted films produced by the method of Geng et al. (2024). The best films were selected based on the highest transparency, the lowest solubility, and the highest antimicrobial properties. Thus, the films were composed of 1 % TG or 1 % PAGEs, with or without 0.5 % MAPs (rich in fucoidan), and with or without 0.5 % zinc oxide. The samples with respective formulations are provided in Table 1. Two control fish fillets were prepared: one without any packaging, termed C1, and another packaged with commercial polytetrafluoroethylene (PTFE) film, termed C2. The PTFE film used had a thickness of approximately 200 μm and a water vapor transmission rate (WVTR) of about 18 g/m^2^·day at 23 °C and 85 % relative humidity, as reported by CMC Klebetechnik GmbH. Three fish fillets were wrapped with films based on PAGE so that one film was composed of 1 % PAGE, 0.5 % MAP, and 0.5 % ZnO (R1), the second film based on PAGE had 1 % PAGE and 0.5 % ZnO (R2), and the third film based on PAGE had 1 % PAGE and 0.5 % MAPs (R3). Similarly, three fish fillets were wrapped using films based on TG so that one film was composed of 1 % TG, 0.5 % MAPs, 0.5 % ZnO (R4), second film based on TG had 1 % TG and 0.5 % ZnO (R5), and third film based on TG had 1 % TG and 0.5 % MAPs (R6). All samples (including controls) were stored in closed, food-grade polyethylene containers to minimize moisture loss and avoid cross-contamination. The containers were placed in a refrigerator maintained at 4 ± 1 °C under dark conditions to prevent light-induced oxidation or microbial effects. Samples were stored separately and evenly spaced within the containers to avoid physical contact and ensure uniform exposure to storage conditions. These conditions were maintained consistently throughout the storage period to ensure comparability between treatments.

**Table 1:** Fish fillet treatments wrapped with non-edible plastic packaging and edible films.

Film components	Runs							
	C1⁎	C2⁎	1	2	3	4	5	6
PAGEs	–	–	1 %	1 %	1 %	–	–	–
TG	–	–	–	–	–	1 %	1 %	1 %
MAPs	–	–	0.5 %	–	0.5 %	0.5 %	–	0.5 %
ZnO	–	–	0.5 %	0.5 %	–	0.5 %	0.5 %	–

⁎ C1 expresses fish fillet without any packaging while C2 indicates fish fillet with PTFE packaging.

### 2.4 Chemical analysis

#### 2.4.1 pH value

The pH of the coated fish fillet samples was measured during 14 days of storage using a digital pH meter (Model: Metrohm 827 pH Lab, Metrohm AG, Herisau, Switzerland) equipped with a combined glass electrode probe (Unitrode with Pt1000, suitable for food applications). For sample preparation, 5 g of fish fillet was homogenized with 45 mL of distilled water using a Philips HR2505/90 home mixer (Philips, Netherlands). The pH of the homogenized mixture was then recorded by immersing the probe directly into the solution (Shakour, Khoshkhoo, Akhondzadeh Basti, Khanjari, & Mahasti Shotorbani, 2021).

#### 2.4.2 Acidity value

The acidity or acidity value (AV) of fish fillet samples was determined based on the titration method during 14 days of storage (Sheybani, Rashidi, Nateghi, Yousefpour and Mahdavi, 2023a, Sheybani, Rashidi, Nateghi, Yousefpour and Mahdavi, 2023b). First, 10 g of samples were taken and mixed with 50 mL of ethanol in an Erlenmeyer. After mixing, 25 mL of mixture were filtered and 3 drops of phenolphthalein solution were added and titrated with 0.1 N NaOH solution. By appearing pink color, the titration was stopped and the volume of consumed basic solution was recorded. Acidity was measured using the relation provided below and expressed as mg KOH per g of fatty acids:

(1) $$\mathrm{Acidity value}=\left[\frac{V\times N\times 56.10}{W}\right]$$

where V indicates the volume of the NaOH solution (mL), N indicates the normality of the NaOH solution, and W implies the weight of the sample (g).

#### 2.4.3 Peroxide value

The peroxide value (PV) of fish fillet samples was determined after the extraction of oil during 14 das of storage (Ei Sheikha et al., 2022; Sheybani, Rashidi, Nateghi, Yousefpour and Mahdavi, 2023a, Sheybani, Rashidi, Nateghi, Yousefpour and Mahdavi, 2023b). For this, 15 g of samples were taken and mixed with 60 mL of methanol, and 60 mL of chloroform and stored overnight. Then, 36 mL of distilled water added to the mixture and the required oil was obtained after 2 h. In a 500 mL- Erlenmeyer, 25 mL of solution containing chloroform and acetic acid at a ratio of 3:2 were mixed with 250 mL of the extracted oil. After, 0.5 mL of saturated potassium iodide solution along with 30 mL of distilled water, and 0.5 mL of starch solution (1 % *w*/w) were added to the provided mixture and the released iodine was amounted by titration with thiosulfate sodium 0.01 N and PV was expressed as meq/kg using the relation provided below:

(2) $$\mathrm{PV}\;\left(\frac{\mathrm{meq}}{\mathrm{kg}}\right)=\frac{\left(S\times N\right)}{W\times 1000}$$

where S and N showcase the volume of titration (mL) and the normality of sodium thiosulfate solution, respectively. W is the weight of the sample (kg).

#### 2.4.4 Total volatile basic nitrogen

The total volatile basic nitrogen (TVB-N) of covered fish fillets was determined during 14 days of storage (Ei Sheikha et al., 2022; Khorami, Babaei, Valizadeh, Naseri, & Golmakani, 2024). Briefly, 10 g of fillet samples were taken and mixed with 2 g of magnesium oxide (MgO) and 300 mL of distilled water in a Kjeldal balloon. The mixture was heated and boiled for 30 min. In an Erlenmeyer 250 mL, 25 mL boric acid 2 % was mixed with some drops of methyl red indicator (0.1 g in 100 mL ethanol). Methyl red can present a red color in acidic media and a yellow color in basic conditions. The distilled extract was collected in a prepared boric acid solution. It should be noted that when the boric acid solution became basic due to the entrance of volatile basic nitrogen compounds, the color was intended to be yellow. The mixture was titrated with sulfuric acid 0.1 N until the color of the boric acid solution became red again. The TVB-N was expressed as mg N/100 g fish fillet. The amount of TVB-N was calculated using the relation presented below:

(3) $$\mathrm{TVB}-N=\frac{\mathrm{the volume of consumed sulfuri acid}\times 1.4}{\mathrm{Weight of sample}}\times 100$$

#### 2.4.5 Thiobarbituric acid reactive substances

Thiobarbituric acid reactive substances (TBARS) were determined based on the colorimetric method during 14 days of storage (Dehghani, Hosseini, Golmakani, Majdinasab, & Esteghlal, 2018; El Sheikha et al., 2022). For this, 200 mg of samples were transferred into 25 mL volumetric balloons and then the volume reached 25 mL using 1-butanol. Then, 5 mL of prepared solution was transferred into tubes and 5 mL TBA indicator was added. TBA indicator was prepared by dissolving 200 mg in 100 mL 1-butanol and the obtained solution was filtered. The tubes were placed in a water bath set at a temperature of 95 °C while the reaction time was 2 h. After, the mixtures were cooled to ambient temperature, and absorbances (As) were read at 530 nm using a UV–vis spectrophotometer. Distilled water was considered as blank (Ab). Therefore, TBARS was expressed based on mg malondialdehyde (MDA)/ kg fish and calculated using the relation provided below:

(4) $$\mathrm{TBARS}=\frac{\left(A_{s}-A_{b}\right)}{200}\times 50$$

### 2.5 Physical analysis

#### 2.5.1 Color

Color characteristics of the fish fillet samples were measured using a smart colorimeter (MAT, 2000 series; IDME Co., Ltd., Shiraz, Iran) using the CIELab color space (Khorami et al., 2024). The instrument was set to an 8 mm aperture opening, diffuse/8° (d/8°) optical geometry, D65 illuminant, and a 10° standard observer angle. The fish fillets were positioned at the sample port, and color parameters L* (lightness), a* (red-green), and b* (yellow-blue) were recorded. Due to the non-uniform color distribution on the fillet surface, measurements were taken at three specific points: the anterior, middle, and posterior sections.

#### 2.5.2 Texture

The texture features including hardness (N), adhesiveness (N.m), springiness (mm), cohesiveness (dimensionless), and chewiness (mJ) of fish fillets were assessed using a texture analyzer (TAXT-2i, Stable Microsystems, Surrey, England) (Khorami et al., 2024). An aluminum cylindrical probe with a diameter of 25 cm was used for two-stage pressure to penetrate to the depth of 50 % at a rate of 2 mm/s so that the distance of two pressures was 30 s.

### 2.6 Microbial analysis

#### 2.6.1 Total bacteria count

Ten g of fish fillets were separated from the internal part of the muscle and mixed with 90 mL of physiological serum and then serial dilutions were provided. One mL of each dilution was taken and poured on the plate count agar (PCA). The cultivated samples were incubated at 37 °C for 24 h over 14 days of storage and then the colonies were enumerated. The results were reported as log CFU/g (Khorami et al., 2024).

#### 2.6.2 Enumeration of *Enterobacteriaceae* Using VRBD Agar

Enterobacteriaceae counts were determined using Violet Red Bile Dextrose (VRBD) agar. Plates were incubated at 30 °C for 24 h, after which colonies were enumerated following the method described by *Improvement of the Shelf Life of Grey Mullet (Mugil cephalus) Fish Steaks Using Edible Coatings Containing Chitosan, Nanochitosan, and Clove Oil During Refrigerated Storage* (Aref et al., 2022).

#### 2.6.3 Antibacterial Activity of Film Extract Against *E. coli and S. aureus*

The colonies of *E. coli* and *S. aureus* were subcultured in Mueller-Hinton Broth (MHB) and incubated at 37 °C for 18–24 h (Sayyari et al., 2021). Bacterial suspensions were prepared according to the 0.5 McFarland standard and further diluted at a 1:300 ratio to achieve a final concentration of approximately 5 × 10^5^ CFU/mL. Then, 50 mg of the film extract was mixed with 1 mL of MHB, and serial dilutions were prepared in 12 tubes, each containing 1 mL of MHB. To each dilution tube, 50 μL of the bacterial suspension was added, and the tubes were incubated at 37 °C for 4 h. Control tubes were also prepared and incubated under the same conditions, including:

• MHB only (negative control),
• MHB with film extract (to assess film sterility),
• MHB with bacterial suspension (positive control for bacterial growth).

#### 2.6.4 Molds and yeasts count

The colonies of molds and yeasts were enumerated for fish fillets during 14 days of storage using YGC agar. Plates were prepared by the pour plate method and then kept under aerobic conditions at 25 °C for 5 days of incubation. After the incubation period, the colonies were enumerated (“Improvement of the shelf life of grey mullet (*Mugil cephalus*) fish steaks using edible coatings containing chitosan, nanochitosan, and clove oil during refrigerated storage (Kazempour-Samak, Rashidi, Ghavami, Sharifan, & Hosseini, 2021).

### 2.7 Statistical analysis

All experiments were conducted in triplicate, and data are presented as means ± standard deviations. A two-way repeated measures ANOVA was used to evaluate the effects of treatment, storage time, and their interaction. When significant differences were found (*P* < 0.05), Duncan's multiple range test was applied for post hoc comparisons. Statistical analyses were performed using SPSS software (version 2022).

## 3 Results and discussion

### 3.1 pH and acidity

The pH and acidity values of fish fillets covered with TG- and PAGE-based films incorporating fucoidan and zinc oxide were monitored over 14 days of storage, with results presented in Table 2. On day 0, pH values ranged from 6.29 to 6.31, with no detectable differences among samples (*p* > 0.05). By day 7, pH values increased across all treatments, ranging from 6.44 to 6.75, indicating a statistically meaningful change (*p* < 0.05). The highest value was obtained for C1 (no coating) and the lowest value was obtained for R4 which was covered with TG-based film incorporated with fucoidan and zinc oxide. Also, there was no observed significant difference between R1 and R4 (*p* > 0.05). pH values were increased on day 14 and the highest values (7.68 and 7.71) were obtained for C1 and C2 (covered with PTFE) and the lowest values (6.79 and 6.72) were obtained for R1 and R4. Results showed that using natural-based nanocomposite films (PAGE and TG) comprising MAPs rich in fucoidan and zinc oxide nanoparticles can remarkably control the pH increase likely due to the liberation of amine compounds through enzymatic and microbial reactions. An increase in pH value intensively occurred in C1 and packaging even with PTFE did not have an impressive effect on pH control. These findings suggest that the application of natural-based nanocomposite films helped to moderate chemical changes in fish fillets during storage, likely due to the antioxidant and antimicrobial properties of TG, PAGE, fucoidan, and zinc oxide nanoparticles. The observed increase in pH may be associated with the breakdown of nitrogenous compounds, a common indicator of spoilage in fish products. In a study, the effects of polylactic acid integrated with nanochitosan composite film with *Ziziphora Clinopodioides*, during the 9 days of storage, pH values were first decreased, and then increased (Shakour et al., 2021). The primary decrease in pH could be due to the activity of lactic acid bacteria and the acidification of the environment (Shakour et al., 2021). The increase in pH value throughout the storage time could be due to the increased generation of volatile nitrogen bases such as ammonia and trimethylamine, likely due to the activity of spoilage bacteria (Shakour et al., 2021). The same results were obtained for coating Salmon with chitosan (Souza et al., 2010). It was reported that the increase in pH might be related to the fast spoilage of the product, with the formation of alkaline autolysis compounds and the production of bacterial metabolites in the muscle during the post-mortem period (Souza et al., 2010). In a study which minced fish meat was fortified with zinc oxide nanoparticles, a lower pH was obtained compared to control on day 3 (Mahato et al., 2024). The addition of nanoparticles proved to be beneficial for the fish stored for a long period. The lower the pH of fish, the lower the chance of microbial and enzymatic reactions (Mahato et al., 2024). Fucoidan coatings have been found as effective packaging system to suppress the mango fruit respiratory (Xu & Wu, 2021). The application of fucoidan coatings significantly inhibited ascorbic acid degradation and minimized weight loss in mango fruits (Xu & Wu, 2021). Indeed, fucoidan coatings decreased decay incidence of mango fruits. The fucoidan coatings efficiently extended the shelf life of mango fruits (Xu & Wu, 2021). The layer-by-layer structure composed of alginate and chitosan containing sulfated fucoidan protected rainbow trout fillets during refrigerated storage (16 days) (Khorami et al., 2024).

**Table 2:** pH and acidity values of fish fillet samples covered by PAGE and TG based films incorporated with fucoidan and ZnO during 14 days of storage.

Treatments	pH			Acidity (mg KOH per g of fatty acids)		
	Day 0	Day 7	Day 14	Day 0	Day 7	Day 14
C1	6.30 ± 0.00^Ca^	6.75 ± 0.01^Ba^	7.68 ± 0.00^Aa^	0.59 ± 0.07^Ca^	2.24 ± 0.05^Ba^	3.79 ± 0.07^Aa^
C2	6.30 ± 0.01^Ca^	6.71 ± 0.00^Ba^	7.71 ± 0.00^Aa^	0.56 ± 0.05^Cab^	2.10 ± 0.10^Ba^	3.55 ± 0.07^Ab^
R1	6.30 ± 0.00^Ca^	6.48 ± 0.00^Bcd^	6.79 ± 0.01^Af^	0.32 ± 0.02^Cde^	0.43 ± 0.08^Bd^	0.79 ± 0.07^Ag^
R2	6.31 ± 0.01^Ca^	6.57 ± 0.01^Bb^	7.02 ± 0.01^Ab^	0.47 ± 0.06^Cb^	0.93 ± 0.10^Bb^	1.82 ± 0.07^Ac^
R3	6.29 ± 0.01^Ca^	6.52 ± 0.00^Bbc^	6.94 ± 0.01^Ac^	0.36 ± 0.02^Ccd^	0.72 ± 0.09^Bbc^	1.31 ± 0.07^Ae^
R4	6.31 ± 0.01^Ca^	6.44 ± 0.00^Bd^	6.72 ± 0.01^Ag^	0.25 ± 0.02^Ce^	0.36 ± 0.02^Bd^	0.51 ± 0.05^Ah^
R5	6.30 ± 0.01^Ca^	6.54 ± 0.00^Bb^	6.83 ± 0.01^Ae^	0.43 ± 0.04^Cbc^	0.81 ± 0.05^Bb^	1.54 ± 0.05^Ad^
R6	6.31 ± 0.00^Ca^	6.49 ± 0.00^Bc^	6.89 ± 0.00^Ad^	0.35 ± 0.02^Ccd^	0.63 ± 0.04^Bc^	1.15 ± 0.08^Af^

Note: Values are expressed as mean ± standard deviation (n = X).

Different uppercase letters in the same row (e.g., within a treatment across days) indicate statistically significant differences between storage days (*p* < 0.05).

Different lowercase letters in the same column (e.g., among treatments on the same day) indicate statistically significant differences between treatments at a given storage time (*p* < 0.05).

Statistical comparisons were made within each variable, and not between different variables (e.g., pH vs. acidity).

C1 expresses fish fillet without any packaging while C2 indicates fish fillet with PTFE packaging.

R1 describes a fish fillet that is coated with a single film made up of 1 % PAGE, 0.5 % MAP, and 0.5 % ZnO; R2 indicates a fish fillet coated with one film consisting of 1 % PAGE and 0.5 % ZnO; R3 refers to a composition of 1 % PAGE and 0.5 % MAPs; R4 includes 1 % TG, 0.5 % MAPs, and 0.5 % ZnO; R5 mentions 1 % TG along with 0.5 % ZnO; R6 outlines a combination of 1 % TG and 0.5 % MAPs.

Regarding the acidity values, the primary values ranged between 0.25 mg KOH/g for R1 and 0.59 mg KOH/g for C1 (*p* < 0.05). On day 7 and day 14, the values were significantly increased for all samples. However, the changes for those covered with TG and PAGE incorporated with fucoidan and zinc oxide nanoparticles were limited. On day 14, the highest value was obtained for C1 (3.79 mg KOH/g) while the lowest value was obtained for R4 (0.51 mg KOH/g). The increase in acid values could be due to production of lactic acid by the activity of lactic acid bacteria or other pathogens produced acid compounds (Kazempour-Samak et al., 2021). As mentioned, the increase in acidity was so limited in samples covered with natural biopolymers composite films comprising zinc oxide nanoparticles and fucoidan compared with samples that were not covered or covered with PTFE. An increase in acidity value could be associated with protein breakdown and the release of phosphoric and lactic acids (Duan, Cherian, & Zhao, 2010). In our study, a limited increase in acidity value and also slight increase in pH values in fish fillets covered by TG and PAGE incorporated with fucoidan and zinc oxide led to pH values adjusted near to 7 which can influence the sensorial characteristics such as odor, color, and texture (Souza et al., 2010).

### 3.2 Peroxide value

**Fig. 1:**
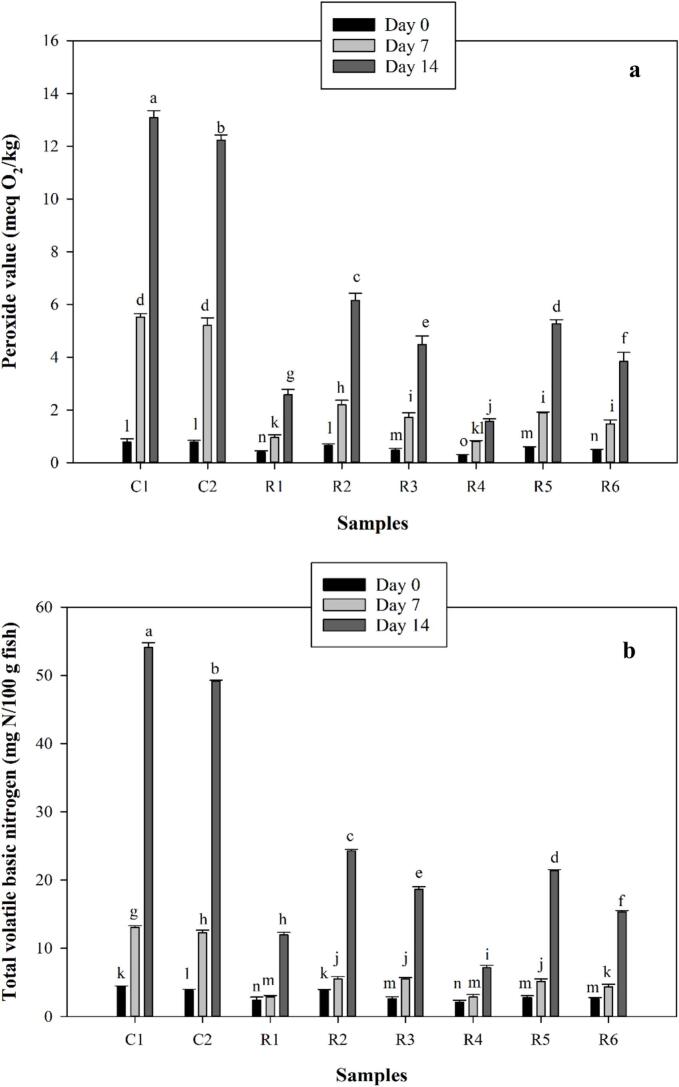

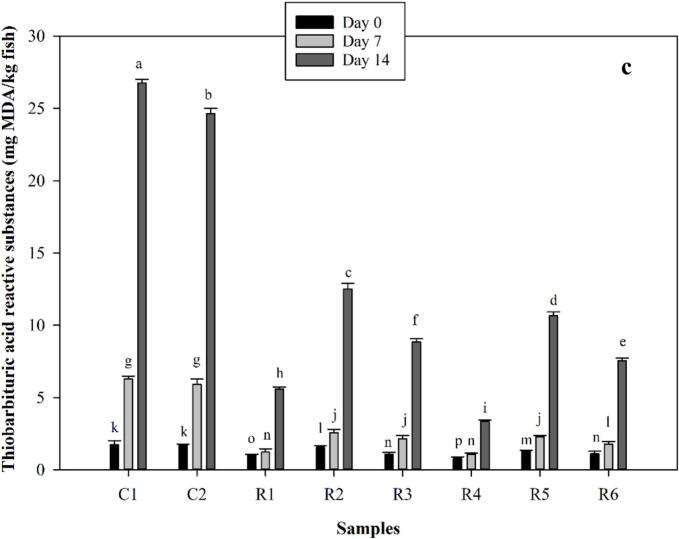
Peroxide value (a), total volatile basic nitrogen (b), and thiobarbituric acid (c) values of covered fish fillets using PAGE and TG incorporated with fucoidan and zinc oxide. Tests were carried out during 14 days of storage. C1 was control fish fillet that was not covered. C2 was control fish fillet that was covered by PTFE. R1 and R4 were fish fillet main samples that were covered by PAGE and TG-based film which were incorporated with fucoidan and zinc oxides, respectively. R2 and R5 were fish fillet samples that were covered by PAGE and TG-based films which were incorporated with only zinc oxides, respectively. R3 and R6 were fish fillet samples that were covered by PAGE and TG-based films which were incorporated with only fucoidan, respectively.

Peroxide value (PV) as the initial oxidation index of fish fillets was determined during 14 days of storage and results were represented in Fig. 1a. PV is an indicator of the initial oxidation level associated with formation of hydroperoxide (Khorami et al., 2024; Shakour et al., 2021). For all samples, PV was significantly increased throughout the storage time (*p* < 0.05). Although the PTFE-coated sample (C2) showed lower peroxide values (PVs) compared to the unpackaged control (C1), both samples had significantly higher PVs than those of the samples coated with biopolymer films that included fucoidan and zinc oxide nanoparticles (*p* < 0.05). The lower PV in C2 indicates that restricting oxygen permeability may help in managing lipid oxidation. While research conducted by Domiszewski, Mierzejewska, Michalska-Pożoga, Rybka, and Rydzkowski (2024) and Gaikwad, Deshmukh, and Lee (2024) revealed that LDPE-based films particularly when enhanced with oxygen-scavenging agents—can mitigate oxidation in fish products, their results are based on synthetic materials like graphene and pyrogallic acid, which are quite different from the natural, marine-derived nanocomposites utilized in this study. Therefore, although the overarching trend of oxygen limitation leading to reduced oxidation is evident, the mechanisms and materials involved are not directly comparable.

In the present study, among the samples covered by the natural biopolymers, the lowest values were found for R1 and R4. Indeed, when PAGE and TG-based films were incorporated with both fucoidan and zinc oxide nanoparticles, the lower PVs were obtained compared to when MAPs rich in fucoidan and zinc oxide nanoparticles were individually added (*p* < 0.05). On another side, fish fillet samples covered by TG-based films showcased significantly lower PVs than those obtained for fish fillet samples covered by PAGE-based films (*p* < 0.05). Based on reports, when rainbow trout fillets were coated with 2 % chitosan and 1.5 % cinnamon oil, PV was efficiently controlled (Ojagh, Rezaei, Razavi, & Hosseini, 2010). It was also reported when zinc oxide nanoparticles were added to the minced fish meat, minimum oxidative changes were obtained only on day 3 not on day 0 and day 6 (Mahato et al., 2024). Various reports have suggested incorporating fucoidan into composite films due to having antimicrobial and antioxidant activities (Jun et al., 2018). Sulfated fucoidan which is extracted from brown seaweeds can serve as a potent antioxidant since it possesses excellent free radical scavenging activity (Khorami et al., 2024; Roy et al., 2024). Both PAGE and TG have carbohydrate structure with various functional groups such as hydroxyl which can donate electrons to free hydroxyl, and hydroperoxides which are primary oxidation products (Pei, Mei, Wu, Yu, & Xie, 2022). Besides, fucoidan with high level of fucose and sulfated structure can render strong antioxidant activity (Bahrami et al., 2025). TG along with sodium alginate formed an efficient coating on large yellow croaker which retarded the initial and secondary oxidation rate (Pei et al., 2022).

### 3.3 Total volatile basic nitrogen

Total volatile basic nitrogen (TVBN) of covered fish fillets was determined during 14 days of storage and results were presented in Fig. 1b. TVBN is related to meat products when they undergo microbial spoilage, resulting in the formation of volatile base compounds such as ammonia, monomethylamine, dimethylamine, and trimethylamine(Khorami et al., 2024). Based on reports, the maximum acceptable TVB-N limitation in fish fillets is around 30 mg N/100 g (Khorami et al., 2024). According to results in Fig. 1b, the TVBN of fish fillets was significantly increased throughout the storage time (*p* < 0.05). The highest values were obtained for C1 and C2 while the latter had lower than the former. Indeed, using PTFE somehow controlled the formation of volatile base nitrogen compounds compared to C1 which was not covered. There are several studies which mentioned efficient impacts of plastics on controlling the oxidation by reduction of initial oxygen concentration. Indeed, presence of a film whether with a natural origin or synthesized origin can control and inhibit the intensive oxidation. On another side, using biocomposite films containing natural biopolymers including PAGEs and TG incorporated with fucoidan and zinc oxide nanoparticles led to a significant reduction of volatile base nitrogen compounds compared to C2 (*p* < 0.05). In previous findings, Palanisamy et al. (2019) specifically investigated the antibacterial efficacy of a fucoidan fraction (Fu-F2) extracted from *Sargassum polycystum*, demonstrating its significant antibacterial activity against *Pseudomonas aeruginosa*, *Staphylococcus aureus*, *Escherichia coli*, and *Streptococcus mutans* (Palanisamy et al., 2019). Controlling microbial spoilage can significantly make obstacles for the formation of volatile base nitrogen compounds. Among the samples, R1 and R4 exhibited significantly lower TVBN than other samples. There was no observed significant difference between the R1 and R4 on days 0 and 7 (*p* > 0.05). The lowest TVBN on day 14 was obtained for R4. Between main samples, the highest values were achieved for R2, R3, and R5 which were remarkably lower than the maximum acceptable level abovementioned. According to previous reports, layer-by-layer coating or bilayer coatings were developed to extend the shelf life of fish fillets. In a study, chitosan, alginate, and fucoidan were used to extend the shelf life of rainbow trout fillets during refrigerated storage (0 to 16 days) (Khorami et al., 2024). It was reported that the fish fillet coated with alginate/fucoidan (1 % *w*/w)-chitosan layer-by-layer coating (AChF1) displayed the lowest TVB-N value. It was declared that the rise in TVB-N could be attributed to the activity of spoilage bacteria and internal enzymes, leading to the production of ammonia and amines (I, II, and III types). Besides, it was elucidated that there was a direct correlation between microbial growth and the TVB-N value. The sample AChF1 exhibited the lowest microbial growth and also displayed the lowest TVB-N value. On the last storage day (16), the control and fish fillet samples coated with only alginate indicated TVB-N values of 32.20 and 30.12 mg N/100 g fish, respectively, which were critical values considering the maximum acceptable level. In contrast, fish fillet samples coated with alginate/fucoidan (0.5 %)-chitosan layer-by-layer coating and alginate-fucoidan (1 %)-chitosan layer-by-layer coating had TVB-N values of 14.28 and 13.44 mg N/100 g fish, respectively (Khorami et al., 2024). Thus, the inclusion of fucoidan in biopolymer coating formulation can efficiently control the formation of volatile base nitrogen compounds.

### 3.4 Thiobarbituric acid reactive substances

Thiobarbituric acid reactive substance (TBARS) is a crucial index in the determination of secondary lipid oxidation products especially malondialdehyde (MDA) which can assess the oxidative deterioration level in food products. The production of MDA is associated with microbial spoilage and oxygen-induced oxidation during the storage time (Mariutti & Bragagnolo, 2022). The values of TBARS were represented in Fig. 1c. As illustrated, the values indicated a rising trend throughout the storage. The highest values were obtained for C1 and C2 with no significant differences on days 0 and 7 (*p* > 0.05) while C2 had a lower TBARS values on the days 14 compared to C1 (*p* < 0.05). Indeed, using PTFE-based packaging system controlled the oxidation by reducing the oxygen accessibility. Using biopolymers included with fucoidan and zinc oxide nanoparticles remarkably lowered the increase in TBARS value during the storage. Among the main samples, R1 and R4 had significantly lower TBARS values than other samples (*p* < 0.05). Especially, R4 which was covered by TG-based nanocomposite containing fucoidan and zinc oxide nanoparticles indicated a lower TBARS value than that obtained for PAGE-based nanocomposite film on the day 14. Based on reports, using chitosan and alginate-based films incorporated with fucoidan efficiently maintained the TBARS values within the acceptable range (Khorami et al., 2024). Accordingly, fish fillets covered by alginate/fucoidan (0.5 and 1 %)-chitosan layer-by-layer coating indicated the lowest TBARS values through the storage (Khorami et al., 2024). The effectiveness of fucoidan in the prevention of lipid oxidation progress could be due to its antioxidant properties. Indeed, MAPs rich in fucoidan that contain hydrogen donor groups such as -COOH and -SO_3_H have been shown to enhance the capacity of scavenging potential (Khorami et al., 2024).

### 3.5 Color

The appearance of food products is a widely accepted indicator of quality and freshness, which can, in turn, influence their nutritional characteristics. In seafood products, particularly fish fillets, instrumental color parameters—namely lightness (L*), redness-greenness (a*), and yellowness-blueness (b*)—are commonly used to assess freshness and potential spoilage. These parameters were measured on day 14 of storage, with results summarized in Table 3. The highest L* values were recorded in groups C1 and C2 (uncoated and PTFE-coated, respectively), suggesting a higher degree of spoilage. Increased L* (lightness) is often associated with protein denaturation, enzymatic degradation, lipid oxidation, and microbial activity, all of which contribute to quality deterioration in fish fillets. Conversely, the lowest L* values were observed in sample R1, followed in ascending order by R3, R2, R4, R6, and R5. Samples coated with PAGE generally exhibited lower L* values than those coated with TG, indicating better color stability and freshness retention. Moreover, the incorporation of fucoidan into the coating matrix had a more pronounced effect in reducing L* values than the addition of zinc oxide nanoparticles (as seen in comparisons between R2 and R3, and R5 and R6). This aligns with the antioxidant and antimicrobial characteristics of fucoidan, which have been documented to inhibit spoilage and maintain the color of fish muscle. The use of bilayer edible coatings made from chitosan, alginate, and fucoidan has demonstrated a significant improvement in the quality of rainbow trout, extending its shelf life by up to 16 days. Furthermore, this approach promotes enhanced preservation of chromaticity (L, a*, b*) while concurrently decreasing microbial loads (Khorami et al., 2024). In terms of a* and b* values, control fillets (C1 and C2) exhibited lower readings than those coated with natural biopolymers, suggesting more advanced deterioration. Declines in a* and b* values are associated with oxidative changes in pigments such as myoglobin, particularly the conversion of red oxymyoglobin to brown metmyoglobin, which leads to darker and less visually appealing fillets (Palanisamy, Singh, Zhang, Zhao, & Benjakul, 2024). These color changes are recognized indicators of spoilage in meat and fish products. Lightness (L*) and related color metrics can be influenced by several intrinsic and extrinsic factors, including pigment concentration, pH, protein denaturation, lipid oxidation, water retention, microbial load, and the muscle's physical structure (Khorami et al., 2024). Khorami et al. (2024) noted that although L* values remained relatively consistent across treatments over time, the a* and b* values exhibited a more pronounced decrease in uncoated samples, thereby emphasizing the importance of biopolymer coatings in preserving color stability and freshness.

**Table 3:** The effects of covering fish fillets with PAGE and TG based films incorporated with fucoidan and zinc oxide on color factors.

Treatments	L*	a*	b*
C1	52.24 ± 0.10^a^	3.75 ± 0.07^g^	10.13 ± 0.02^h^
C2	51.71 ± 0.04^b^	3.87 ± 0.10^g^	10.35 ± 0.02^g^
R1	44.76 ± 0.11^h^	6.83 ± 0.04^a^	13.23 ± 0.05^a^
R2	46.29 ± 0.09^f^	6.20 ± 0.08^c^	12.63 ± 0.06^c^
R3	45.43 ± 0.19^g^	6.56 ± 0.08^b^	12.99 ± 0.07^b^
R4	48.63 ± 0.19^e^	5.22 ± 0.08^d^	11.67 ± 0.11^d^
R5	50.66 ± 0.11^c^	4.31 ± 0.09^f^	10.89 ± 0.15^f^
R6	49.63 ± 0.11^d^	4.80 ± 0.04^e^	11.21 ± 0.02^e^

Different small superscript letters indicate differences between values within a column (*P* < 0.05).

C1 expresses fish fillet without any packaging while C2 indicates fish fillet with PTFE packaging.

R1 describes a fish fillet that is coated with a single film made up of 1 % PAGE, 0.5 % MAP, and 0.5 % ZnO; R2 indicates a fish fillet coated with one film consisting of 1 % PAGE and 0.5 % ZnO; R3 refers to a composition of 1 % PAGE and 0.5 % MAPs; R4 includes 1 % TG, 0.5 % MAPs, and 0.5 % ZnO; R5 mentions 1 % TG along with 0.5 % ZnO; R6 outlines a combination of 1 % TG and 0.5 % MAPs.

### 3.6 Texture

Texture properties of uncoated and coated fish fillets were determined and results were provided in Table 4. Regarding the hardness, the highest value was obtained for R4 which was coated with TG and incorporated with fucoidan and zinc oxide nanoparticles. R2 and R3 also showed significantly higher hardness values than the other samples (*p* < 0.05). However, the simultaneous inclusion of fucoidan and nanoparticles into the PAGE coating appeared to reduce the hardness compared to when each was added individually. Indeed, when fucoidan and nanoparticles were added to PAGE individually (R2 and R3), higher hardness values were obtained compared to R1. In contrast, the simultaneous addition of fucoidan and zinc oxide nanoparticles to the TG coating (R4) resulted in a higher hardness value compared to R5 and R6, where only one additive was used. These findings suggest that the combined presence of fucoidan and nanoparticles in the TG matrix may enhance the firmness of the fillets. The lowest hardness values were observed for R5 and R6. Regarding adhesiveness, the highest values were recorded for R4 and R5, which were coated with the TG-based formulation and incorporated with either both additives (R4) or only nanoparticles (R5). Further, it should be noted that most of the main samples covered by PAGE and TG indicated slightly higher adhesiveness values compared to C1 and C2. In terms of springiness, the highest values were obtained for R3, followed by R4 and R5. Also, R4, R5, and R3 indicated higher values of cohesiveness. Regarding the chewiness, the highest values were obtained for R3, followed by R4 and R2.

**Table 4:** The effects of covering fish fillets using PAGE and TG based films incorporated with fucoidan and zinc oxide on texture properties.

Treatments	Hardness (N)	Adhesiveness (N.m)	Springiness (mm)	Cohesiveness (dimensionless)	Chewiness (mJ)
C1	11.71 ± 0.48^e^	0.0044 ± 0.000^f^	17.16 ± 0.96^f^	0.15 ± 0.00^e^	30.88 ± 4.07^f^
C2	13.11 ± 0.39^d^	0.0047 ± 0.000^d^	18.16 ± 0.97^d^	0.17 ± 0.00^d^	40.32 ± 3.45^e^
R1	12.77 ± 0.49^d^	0.0046 ± 0.000^e^	18.76 ± 0.58^d^	0.16 ± 0.01^d^	39.88 ± 5.72^ef^
R2	17.15 ± 0.24^c^	0.0051 ± 0.000^c^	20.86 ± 0.35^c^	0.25 ± 0.00^c^	89.53 ± 2.22^c^
R3	17.83 ± 0.26^b^	0.0052 ± 0.000^b^	24.73 ± 0.32^a^	0.27 ± 0.01^ab^	118.68 ± 4.84^a^
R4	18.28 ± 0.37^a^	0.0054 ± 0.000^a^	22.20 ± 0.88^b^	0.28 ± 0.00^a^	115.05 ± 8.22^b^
R5	8.86 ± 0.42^f^	0.0054 ± 0.000^a^	22.76 ± 0.40^b^	0.29 ± 0.01^a^	59.36 ± 5.76^d^
R6	8.45 ± 0.20^f^	0.0044 ± 0.000^f^	18.83 ± 0.15^d^	0.16 ± 0.01^d^	26.56 ± 2.57^g^

Different small superscript letters indicate differences between values within a column (*P* < 0.05).

C1 expresses fish fillet without any packaging while C2 indicates fish fillet with PTFE packaging.

R1 describes a fish fillet that is coated with a single film made up of 1 % PAGE, 0.5 % MAP, and 0.5 % ZnO; R2 indicates a fish fillet coated with one film consisting of 1 % PAGE and 0.5 % ZnO; R3 refers to a composition of 1 % PAGE and 0.5 % MAPs; R4 includes 1 % TG, 0.5 % MAPs, and 0.5 % ZnO; R5 mentions 1 % TG along with 0.5 % ZnO; R6 outlines a combination of 1 % TG and 0.5 % MAPs.

### 3.7 Microbial count

Total bacterial and *Enterobacteriaceae* counts of coated and uncoated fish fillets were assessed over 14 days of storage (Table 5a). As expected, both microbial groups showed increasing trends during refrigerated storage. However, coated samples—particularly those treated with nanocomposite films—demonstrated significantly lower microbial loads compared to uncoated controls. Among the samples, R1 and R4 exhibited the lowest bacteria and *Enterobacteriaceae* counts which were comparable with control fish fillets (*p* < 0.05). It was observed that when TG-based films efficiently controlled the growth of bacteria compared to those of PAGE-based films. Also, it was found that the inclusion of both fucoidan and nanoparticles efficiently prevented the bacteria growth compared to the individual inclusion of fucoidan and nanoparticles. Besides, no *Enterobacteriaceae* were detected for fish fillets covered by PAGE and TG biopolymers on day 0 and 0.33 log CFU/g was only detected for R6 on day 7. On day 14, R1 and R4 did not have *Enterobacteriaceae* while R2 and R5 indicated *Enterobacteriaceae* which were significantly lower than those obtained for C1 and C2 on day 7 (*p* < 0.05). Therefore, using PAGE and TG-based films incorporated with fucoidan and zinc oxide nanoparticles efficiently controlled bacteria growth of fish fillets during long storage periods.

**Table 5a:** Total bacteria and *Enterobacteriaceae* counts of covered fish fillets using PAGE and TG based films incorporated with fucoidan and zinc oxide.

Treatments	Total bacteria count (log CFU/g)			*Enterobacteriaceae* count (log CFU/g)		
	Day 0	Day 7	Day 14	Day 0	Day 7	Day 14
C1	3.67 ± 0.45^Ca^	6.78 ± 0.17^Ba^	7.85 ± 0.21^Ab^	4.00 ± 0.00^Ca^	8.00 ± 2.64^Ba^	10.20 ± 1.52^Aa^
C2	3.67 ± 0.06^Ca^	6.45 ± 0.35^Ba^	7.14 ± 0.32^Ac^	4.66 ± 3.05^Cb^	8.00 ± 3.60^Ba^	10.45 ± 1.08^Aa^
R1	2.56 ± 0.09^Cd^	3.27 ± 0.36^Be^	5.26 ± 0.04^Af^	<[detection limit]^Ac^	0.00 ± 0.00^Ad^	0.00 ± 0.00^Af^
R2	3.41 ± 0.14^Cb^	5.66 ± 0.45^Bb^	8.08 ± 0.16^Aa^	<[detection limit]^Cc^	0.66 ± 0.57^Bb^	3.66 ± 1.52^Ab^
R3	2.67 ± 0.20^Cd^	4.92 ± 0.37^Bc^	6.59 ± 0.11^Ad^	<[detection limit]^Ac^	<[detection limit]^Ad^	1.33 ± 0.05^Ad^
R4	2.23 ± 0.18^Ce^	2.99 ± 0.11^Be^	4.42 ± 0.04^Ag^	<[detection limit]^Ac^	<[detection limit]^Ad^	0.00 ± 0.00^Af^
R5	3.01 ± 0.07^Cc^	5.18 ± 0.18^Bb^	7.28 ± 0.09^Ac^	<[detection limit]^Bc^	<[detection limit]^Bd^	2.33 ± 0.57^Ac^
R6	2.71 ± 0.22^Cd^	4.27 ± 0.31^Bd^	6.09 ± 0.06^Ae^	0.00 ± 0.00^Cc^	0.33 ± 0.05^Bc^	1.00 ± 0.02^Ae^

Values are expressed as mean ± standard deviation. Different lowercase superscript letters in the same column indicate significant differences among treatments on the same day. Different uppercase superscript letters in the same row indicate significant differences within the same treatment over the storage period (*P* < 0.05).

C1 expresses fish fillet without any packaging while C2 indicates fish fillet with PTFE packaging.

R1 describes a fish fillet that is coated with a single film made up of 1 % PAGE, 0.5 % MAP, and 0.5 % ZnO; R2 indicates a fish fillet coated with one film consisting of 1 % PAGE and 0.5 % ZnO; R3 refers to a composition of 1 % PAGE and 0.5 % MAPs; R4 includes 1 % TG, 0.5 % MAPs, and 0.5 % ZnO; R5 mentions 1 % TG along with 0.5 % ZnO; R6 outlines a combination of 1 % TG and 0.5 % MAPs.

*E. coli* and *S. aureus* counts of covered fish fillets were assessed and results were provided in Table 5b. Accordingly, no *E. coli* colonies were detected for control fish fillets and main samples through, the storage time. Regarding *S. aureus* count, only control fish fillets (C1 and C2) indicated 3.33 and 4 log CFU/g on day 7 and no colonies were detected for the main samples. On day 14, R1 and R4 indicated no colonies while the highest values were obtained for R2 and R5. It was found that when both fucoidan and zinc oxide nanoparticles were incorporated into PAGE and TG-based films, efficient antimicrobial properties were observed.

**Table 5b:** *E. coli* and *S. aureus* counts of covered fish fillets using PAGE and TG based films incorporated with fucoidan and zinc oxide.

Treatments	*E. coli* (log CFU/g)			*S. aureus* (log CFU/g)		
	Day 0	Day 7	Day 14	Day 0	Day 7	Day 14
C1	<[detection limit]^Aa^	<[detection limit]^Aa^	<[detection limit]^Aa^	<[detection limit]^Ca^	3.33 ± 1.52^Ba^	5.14 ± 0.62^Aa^
C2	<[detection limit]^Aa^	<[detection limit]^Aa^	<[detection limit]^Aa^	<[detection limit]^Ca^	4.00 ± 1.73^Ba^	5.74 ± 0.41^Aa^
R1	<[detection limit]^Aa^	<[detection limit]^Aa^	<[detection limit]^Aa^	<[detection limit]^Aa^	<[detection limit]^Ac^	0.00 ± 0.00^Ad^
R2	<[detection limit]^Aa^	<[detection limit]^Aa^	<[detection limit]^Aa^	<[detection limit]^Ba^	<[detection limit]^Bc^	1.33 ± 0.57^Ab^
R3	<[detection limit]^Aa^	<[detection limit]^Aa^	<[detection limit]^Aa^	<[detection limit]^Ba^	<[detection limit]^Bc^	0.66 ± 0.02^Ac^
R4	<[detection limit]^Aa^	<[detection limit]^Aa^	<[detection limit]^Aa^	<[detection limit]^Aa^	<[detection limit]^Ac^	0.00 ± 0.00^Ad^
R5	<[detection limit]^Aa^	<[detection limit]^Aa^	<[detection limit]^Aa^	<[detection limit]^Ba^	<[detection limit]^Bc^	1.66 ± 0.57^Ab^
R6	<[detection limit]^Aa^	<[detection limit]^Aa^	<[detection limit]^Aa^	<[detection limit]^Aa^	<[detection limit]^Ac^	<[detection limit]^Ad^

Values are expressed as mean ± standard deviation. Different lowercase superscript letters in the same column indicate significant differences among treatments on the same day. Different uppercase superscript letters in the same row indicate significant differences within the same treatment over the storage period (P < 0.05).

C1 expresses fish fillet without any packaging while C2 indicates fish fillet with PTFE packaging.

R1 describes a fish fillet that is coated with a single film made up of 1 % PAGE, 0.5 % MAP, and 0.5 % ZnO; R2 indicates a fish fillet coated with one film consisting of 1 % PAGE and 0.5 % ZnO; R3 refers to a composition of 1 % PAGE and 0.5 % MAPs; R4 includes 1 % TG, 0.5 % MAPs, and 0.5 % ZnO; R5 mentions 1 % TG along with 0.5 % ZnO; R6 outlines a combination of 1 % TG and 0.5 % MAPs.

In terms of mold and yeast counts, rising trends were observed for control fish fillets and those were covered by PAGE and TG during the storage (Table 5c). On day 14, the highest count was obtained for C1 and C2 and the lowest count was obtained for R1 and R4. On day 14, R1 and R4 indicated the lowest mold and yeast counts while R4 indicated lower than R1, which could be attributed to stronger anti-mold and anti-yeast activity of TG-based nanocomposite film. Among the tested formulations, the TG-based nanocomposite film showed a stronger inhibitory effect on microbial growth, including yeasts and molds. This could be attributed to the synergistic antimicrobial action of its active components (fucoidan and ZnO nanoparticles) and the barrier properties of the film itself. Similar antimicrobial effects were reported by El Sheikha et al. (2022a), where Mackerel Tuna fillets coated with xanthan–propolis composite films exhibited reduced fungal growth and improved microbial stability during cold storage. Based on the literature, the bactericidal pathway of fucoidans was attributed to the destruction of the cytomembranes and the target molecules are the membrane proteins, which can result in changed membrane fluidity, increased permeability, and ultimately cell lysis and/or activated autophagocytosis (Liu et al., 2017). A study on fucoidans from *Laminaria hyperborea* highlights inhibitory effects against Gram-negative and Gram-positive bacteria. These include mechanisms like cell wall synthesis disruption, inhibition of adhesion and biofilm formation, and alteration of membrane fluidity or integrity. Specifically, depoylmerized fucoidans (<6 kDa) from *Laminaria japonica* have shown bacteriostatic and bacteriocidal effects against *E. coli* and *S. aureus* (Beagan et al., 2024). The inclusion of zinc oxide nanoparticles (ZnO-NPs) in the coating also played a crucial role, as these nanoparticles are well-documented for their strong antibacterial, antifungal, and UV-blocking properties. ZnO-NPs disrupt microbial membranes and generate reactive oxygen species (ROS), leading to oxidative stress and bacterial death (Sirelkhatim et al., 2015). Together, these findings demonstrate that the developed nanocomposite films effectively slowed microbial spoilage and extended the microbial shelf life of Beluga fillets.

**Table 5c:** *Mold* and *yeast* count of covered fish fillets using PAGE and TG based films incorporated with fucoidan and zinc oxide.

Treatments	*Mold* and *yeast* (log CFU/g)		
	Day 0	Day 7	Day 14
C1	1.89 ± 0.24^Cab^	3.49 ± 0.07^Ba^	4.58 ± 0.14^Aa^
C2	1.99 ± 0.08^Ca^	3.30 ± 0.13^Ba^	4.74 ± 0.15^Aa^
R1	1.34 ± 0.08^Cd^	1.78 ± 0.16^Be^	2.73 ± 0.09^Ae^
R2	1.78 ± 0.11^Cb^	2.85 ± 0.23^Bb^	4.12 ± 0.12^Ab^
R3	1.45 ± 0.13^Cc^	2.58 ± 0.22^Bbc^	3.36 ± 0.08^Ad^
R4	1.25 ± 0.13^Cd^	1.60 ± 0.14^Be^	2.31 ± 0.08^Af^
R5	1.62 ± 0.08^Cbc^	2.65 ± 0.05^Bc^	3.76 ± 0.14^Ac^
R6	1.46 ± 0.09^Cc^	2.24 ± 0.13^Bd^	3.20 ± 0.07^Ad^

Different small superscript letters indicate differences between values within a column (*P* < 0.05).

C1 expresses fish fillet without any packaging while C2 indicates fish fillet with PTFE packaging.

R1 describes a fish fillet that is coated with a single film made up of 1 % PAGE, 0.5 % MAP, and 0.5 % ZnO; R2 indicates a fish fillet coated with one film consisting of 1 % PAGE and 0.5 % ZnO; R3 refers to a composition of 1 % PAGE and 0.5 % MAPs; R4 includes 1 % TG, 0.5 % MAPs, and 0.5 % ZnO; R5 mentions 1 % TG along with 0.5 % ZnO; R6 outlines a combination of 1 % TG and 0.5 % MAPs.

## 4 Conclusions

Beluga fish fillets were successfully coated with PAGE and TG films incorporated with fucoidan and zinc oxide nanoparticles. These natural-based coatings significantly preserved the quality of fish fillets compared to PTFE packaging. Specifically, the PAGE and TG coatings helped control increases in acidity and pH, while limiting oxidative spoilage indicators such as peroxide value (PV), thiobarbituric acid reactive substances (TBARS), and total volatile basic nitrogen (TVBN). The incorporation of fucoidan notably enhanced the antioxidant activity of the films, improving oxidative stability. Moreover, microbial analysis showed a substantial reduction in bacterial growth, with no detection of *E. coli*, Enterobacteriaceae, or *S. aureus* in treated samples. These findings suggest that PAGE and TG coatings enriched with natural additives like fucoidan and zinc oxide nanoparticles can serve as effective packaging solutions for PUFA-rich seafood like Beluga fish, maintaining both nutritional quality and microbial safety. The use of such nanocomposite films is promising for extending shelf life and ensuring food safety.

However, this study was limited to laboratory-scale experiments under controlled storage conditions and focused on a single fish species. Future research should explore the scalability of film production, sensory impacts of coatings on consumer acceptability, and their effectiveness across different types of fish or other high-value food products. Additionally, long-term environmental safety and biodegradability of these films should be assessed. The cultivation of seaweed species rich in marine algal polysaccharides (MAPs) such as fucoidan could also support sustainable large-scale production of bioactive packaging materials.

## CRediT authorship contribution statement

**Sahba Bahrami Freadooni:** Writing – original draft, Software, Investigation. **Leila Nateghi:** Writing – review & editing, Supervision, Methodology. **Ladan Rashidi:** Writing – review & editing, Supervision, Conceptualization.

## Funding

All authors declare that no funds and grants were received during this research.

## Declaration of competing interest

The authors declare that they have no known competing financial interests or personal relationships that could have appeared to influence the work reported in this paper.

## Data Availability

Data will be made available on request.
